# Management and Incidence of *Enterobius vermicularis* Infestation in Appendectomy Specimens: A Cross-Sectional Study of 6359 Appendectomies

**DOI:** 10.3390/jcm13113198

**Published:** 2024-05-29

**Authors:** Zenon Pogorelić, Vlade Babić, Marko Bašković, Vladimir Ercegović, Ivana Mrklić

**Affiliations:** 1 Department of Surgery, School of Medicine, University of Split, 21000 Split, Croatia; 2 Department of Pediatric Surgery, University Hospital of Split, 21000 Split, Croatia; 3 Department of Pediatric Surgery, Children’s Hospital Zagreb, 10000 Zagreb, Croatia; 4 Scientific Centre of Excellence for Reproductive and Regenerative Medicine, School of Medicine, University of Zagreb, 10000 Zagreb, Croatia; 5 Department of Pathology, Forensic Medicine and Cytology, University Hospital of Split, 21000 Split, Croatia; 6 Department of Pathology, School of Medicine, University of Split, 21000 Split, Croatia

**Keywords:** *Enterobius vermicularis*, enterobiasis, acute appendicitis, appendectomy, vermiform appendix, children

## Abstract

**Background:** The role of *Enterobius vermicularis* infestation in the context of appendicitis is largely overlooked, but *Enterobius vermicularis* is considered an unexpected and significant appendicectomy finding. The aim of this study was to investigate the frequency of *Enterobius vermicularis* findings in appendectomies and to evaluate the clinical and histopathologic features of patients with *Enterobius vermicularis*-associated acute appendicitis and those with appendiceal *Enterobius vermicularis* infestation. **Methods:** The medical records of all children who underwent an appendectomy in two large pediatric centers in Croatia between 1 January 2009 and 1 January 2024 were retrospectively reviewed. Of 6359 appendectomies, 61 (0.96%) children were diagnosed with *Enterobius vermicularis* on histopathology and included in further analysis. The groups were compared with regard to demographic characteristics, laboratory values, clinical features and histopathological findings. **Results**: The incidence of enterobiasis fluctuated slightly in the individual study years, but was constant overall. The median age of all patients was 11 years (IQR 8.5, 13), with females predominating (60.7%). Acute appendicitis was observed in 34% of the appendiceal species. The patients with *Enterobius vermicularis* infestation, without appendicitis, were younger (9 years (IQR 8, 13) vs. 12 years (IQR 10, 15); *p* = 0.020), had longer duration of symptoms (36 h (IQR, 12, 48) vs. 24 h (IQR, 12, 36); *p* = 0.034), lower body temperature (37 °C (IQR 36.8, 37.4) vs. 37.6 °C (IQR, 37, 38.6) *p* = 0.012), lower Appendicitis Inflammation Response (AIR) score (3 (IQR 2, 5) vs. 7 (IQR 5, 9.5) *p* < 0.001), lower incidence of rebound tenderness (57.1% vs. 20%; *p* = 0.003) and less frequent vomiting (12.5% vs. 47.6%; *p* = 0.004) compared to the patients with *Enterobius vermicularis*-associated acute appendicitis. Acute inflammatory markers in the laboratory showed significantly higher values in the group of patients with acute appendicitis: C-reactive protein (*p* = 0.009), White blood cells (*p* = 0.001) and neutrophils (*p* < 0.001). Eosinophilia was not found in any of the groups, although eosinophil counts were significantly higher in children who had *Enterobius vermicularis* infestation than in those with *Enterobius vermicularis*-related appendicitis (2.5% (IQR 0.9, 4.3) vs. 1.8% (IQR 0.7, 2.1); *p* = 0.040). **Conclusions**: Pediatric surgeons should consider *Enterobius vermicularis* infestation as a differential diagnosis when removing a vermiform appendix. Younger age, longer duration of symptoms, lower body temperature, lower AIR score, lower diameter of the appendix and normal laboratory inflammatory markers could predict *Enterobius vermicularis* infection in children presenting with right iliac fossa pain and avoid unnecessary appendectomy.

## 1. Introduction

Acute appendicitis is still considered the most common cause of abdominal pain in the entire population, occurring in about 1:1000 people per year [1,2,3]. Although appendicitis is a common diagnosis in the pediatric population, the exact cause of most acute cases of appendicitis is still unknown [4]. Other common cases of acute appendicitis include luminal obstruction with lymphoid hyperplasia or fecaliths; rarely, acute appendicitis is caused by appendiceal tumors, parasitic infestations or foreign bodies [3,4]. The most common treatment of choice for appendicitis is appendectomy [1,2,3,4,5,6]. Appendectomy has a lifetime risk of about 7%, making it one of the most commonly performed surgical procedures in the world [2,7]. Despite advances in medical diagnostics, the average rate of negative appendectomies is still 10–15% [2]. Recently, conservative treatment with antibiotics has become increasingly popular as an adequate alternative to surgical approaches to acute appendicitis, although it carries the risk of overlooking some other serious diagnoses such as neuroendocrine tumors [8,9].

*Enterobius vermicularis*, also known as pinworm or *Oxyuris vermicularis*, is a common parasite found in nearly 200 million people around the world [7,10,11,12,13,14,15]. Infection with *Enterobius vermicularis* is prevalent worldwide regardless of geographic and socioeconomic circumstances, with a higher incidence in the pediatric population and people living in crowded residential areas [10,13,14]. Enterobiasis, a parasitic infection caused by the pinworm *Enterobius vermicularis*, is characterized by symptoms such as perianal itching, insomnia, restlessness, loss of appetite, weight loss, irritability, stomach pain and nausea, and it can manifest as vulvovaginitis, ileocolitis, mesenteric abscess, salpingitis and appendicitis or be asymptomatic [14,15]. In addition, enterobiasis has been shown to cause the formation of granulomas in the kidney, peritoneal cavity, female genital tract, male urinary tract and appendix, often leading to misdiagnosis of the disease itself [7,15,16,17,18,19,20,21].

The role of parasitic infections, particularly *Enterobius vermicularis*, in the context of appendicitis, is largely overlooked, but *Enterobius vermicularis* is considered an unexpected and significant appendectomy finding [7,10,12,13]. Histopathologic studies of appendicitis cases show the presence of *Enterobius vermicularis* in the lumen and wall of the appendix in association with a marked increase in inflammation of the appendix [21]. This raises interesting questions about the mechanisms by which *Enterobius vermicularis* infestation contributes to the inflammatory cascade that leads to acute appendicitis. Despite these findings, the correct mechanisms underlying the possible link between enterobiasis and appendicitis are still speculative. Hypotheses range from obstruction of the lumen by the movement of the pinworm to the triggering of an immune response that contributes to inflammation [7,22,23]. Adult pinworms attach to the wall of the cecum, appendix and adjacent intestinal segments. Female pinworms are fertilized in the intestine and then migrate to the colon, rectosigmoid or anal cavity, where they deposit eggs and die [14,24]. The eggs deposited by female pinworms often cause itching, which is why they are frequently found under the fingernails of infected patients and in dust samples from households with infected relatives and family members [14,24]. Further research is crucial to decipher these mechanisms and gain a clearer understanding of parasitic involvement in appendiceal pathology. However, recent studies have also shown that *Enterobius vermicularis* has a major impact on the gut microbiome, affecting the composition of the host microbiome and leading to changes in important functions of intestinal epithelial health, metabolism and adaptive and innate immune responses [24]. The most common non-surgical treatment for *Enterobius vermicularis* infection is the use of antihelminthics such as albendazole and mebendazole, both of which belong to the benzimidazole class and inhibit microtubule formation in parasitic organisms, effectively treating pinworms and other parasitic infections [25,26].

The prognosis for *Enterobius vermicularis* infections is considered excellent, and patients are advised to check in with their physicians after completing treatment to make sure there is no reinfection, as the recurrence of symptoms will lead to further testing and new treatment options [14,25]. However, as *Enterobius vermicularis* has an easy route of transmission, reinfection remains the main cause of parasite infection development. As the full life cycle of the helminths from egg to adult worm takes 2 to 4 weeks, reinfection is easily possible [15]. Because it is a disease with a very good prognosis, pinworm infection is treated in most cases by a general practitioner, with patients themselves playing a role in treatment by cleaning their hands and clothing with hot water and soap and taking other measures to prevent reinfection caused by ingesting pinworm eggs as a result of not cleaning their hands [10,25]. Apart from *Enterobius vermicularis*, parasites such as *Ascaris lumbricoides, Taenia saginata, Giardia* spp. or *Entamoeba histolytica* are very rarely mentioned as a cause of acute appendicitis and are usually the result of a high intestinal parasite load [27,28].

The aim of this study was to investigate the incidence, clinical outcome and histopathologic features of *Enterobius vermicularis* findings in appendectomies.

## 2. Methods

### 2.1. Patients

**Figure 1:**
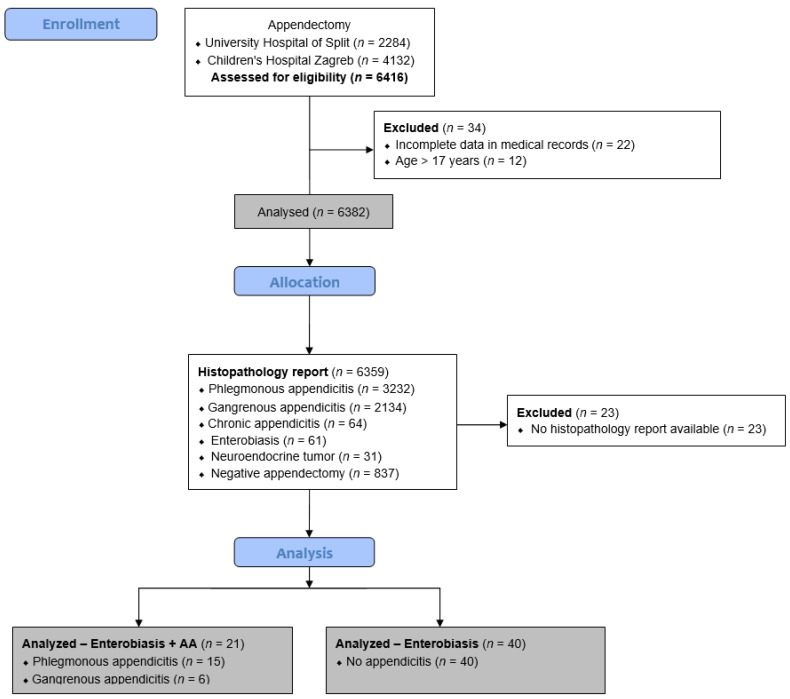
Flowchart of the study. AA—Acute appendicitis.

A cross-sectional study was conducted in two centers in Croatia: Department of Pediatric Surgery, University Hospital of Split, and Department of Pediatric Surgery, Children’s Hospital Zagreb from 1 January 2009 to 1 January 2024. During the selected study period, a total of 6359 appendectomies were performed in both centers. Of these, 61 children (0.96%) were diagnosed with *Enterobius vermicularis* infestation of the appendix and included in the study. The pediatric patients who had undergone appendectomy for suspected acute appendicitis at both institutions and had been histopathologically diagnosed with *Enterobius vermicularis* infestation of the appendix were included in the study. Patients older than 17 years, patients who had undergone conservative treatment for acute appendicitis, patients for whom no histopathologic report was available, patients with a follow-up period of less than 3 months or patients with incomplete data in medical records were excluded from the study (Figure 1).

### 2.2. Institutional Review Board Statement

The study was conducted in accordance with the Declaration of Helsinki of the World Medical Association, and the Institutional Review Board of our hospital approved the study (Approval number: 520-03/23-01/259; Date of approval: 29 January 2024).

### 2.3. Outcomes of the Study

The primary endpoint of the present study was the frequency and treatment outcome of patients diagnosed with *Enterobius vermicularis* infestation of the appendix. Secondary endpoints included differences between the demographic data, laboratory inflammatory markers, clinical features and pathohistological findings of patients with *Enterobius vermicularis*-associated acute appendicitis and those with *Enterobius vermicularis* infestation without appendicitis.

### 2.4. Data Collection and Study Design

**Figure 2:**
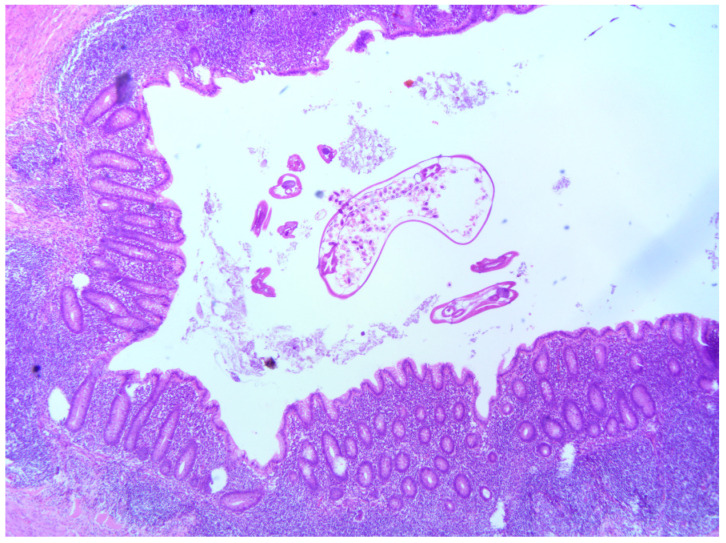
Acute appendicitis with luminal *Enterobius vermicularis* in a 13-year-old girl, HE, 4×. Neutrophilic infiltration in all layers of the appendix wall with *Enterobius vermicularis* in the lumen of the appendix.

The following data were collected for each patient included in the study: baseline demographic characteristics (age, gender, comorbidities, weight, height and body mass index—BMI); preoperative laboratory values—white blood cell (WBC) count, a differential blood count (neutrophils, lymphocytes, monocytes, eosinophils and basophils) and C-reactive protein (CRP); and clinical findings—body temperature, presence of right lower quadrant pain and/or rebound tenderness, presence of nausea/vomiting and duration of onset of symptoms. In addition, the Appendicitis Inflammation Response (AIR) score was calculated for each patient included in the study [29]. All patients underwent open or laparoscopic appendectomy. Both techniques have been described in our previous publications [6,30,31]. For each patient, the histopathology report was reviewed for the presence of *Enterobius vermicularis* infestation (Figure 2). Based on the histopathology report, the patients with *Enterobius vermicularis* infestation were divided into two groups. The first study group (acute appendicitis) included the patients with *Enterobius vermicularis* infestation associated with inflammation of the appendix. Regarding findings on histopathology, the degree of inflammation of the appendix was interpreted as phlegmonous, gangrenous or chronic inflammation [2,4]. The second study group (non-inflamed appendix) consisted of patients with *Enterobius vermicularis* infestation of the appendix but without inflammation of the appendix. For each patient, the length of hospital stay, the reason for appendectomy (elective or urgent), the type of surgery (laparoscopic or open), complications and follow-up care were investigated.

### 2.5. Postoperative Treatment and Follow-Up

Children were discharged from the hospital if they tolerated oral intake and were free of symptoms and fever. All children aged two years or older, in whom the histopathology report revealed *Enterobius vermicularis*, received mebendazole (Vermox, FAMAR SA, Athens, Greece) in a single dose of 100 mg, which was repeated after 14 days. Seven and 30 days after discharge, follow-up examinations were performed in the outpatient polyclinic to detect early or late complications.

### 2.6. Statistical Analysis

The Statistical Package for the Social Sciences (SPSS) 28.0 (IBM Corp., Armonk, NY, USA) and Microsoft Excel for Windows Version 11.0 (Microsoft Corporation, Redmond, WA, USA) were used for statistical analysis of the data. The distribution of quantitative data was expressed as median and interquartile range (IQR). Absolute numbers and percentages were used to describe categorical data. For comparison of continuous variables the Mann–Whitney U test was used, while the chi-squared test was used to compare categorical variables. A two-tailed Fisher’s exact test was used when the frequency of events in a particular cell was low. All *p*-values < 0.05 were considered significant.

## 3. Results

### 3.1. Incidence of Enterobiasis among Patients Who Underwent Appendectomy

During the 15-year study period, a total of 6359 appendectomies were performed in pediatric patients because of suspected acute appendicitis at two pediatric centers. Of these, 61 (0.96%) patients were diagnosed with *Enterobius vermicularis*-related appendicitis. The incidence of enterobiasis fluctuated slightly in the individual study years, but was constant overall. Acute and chronic appendicitis were diagnosed histopathologically in 5366 (84.38%) and 64 (1.00%) children, respectively. A neuroendocrine tumor was found in 31 (0.49%) histopathological specimens. The negative appendectomy rate in this study was 13.16%. Table 1 summarizes the histopathological findings of all patients included in the study.

### 3.2. Demographic Characteristics and Clinical Data of the Patients with Appendiceal Enterobiasis

The median age of all patients was 11 (IQR 8.5, 13) years, with females predominating (*n* = 37, 60.7%). The median duration of symptoms was 24 h (IQR 12, 36), and the median body temperature was 37.4 °C (IQR 36.8, 38.1). Acute abdominal pain occurred in the majority of patients (*n* = 57; 93.4%), while abdominal guarding was observed in 20 patients (32.8%). The median AIR score was 5 (IQR 2, 7).

The patients with *Enterobius vermicularis* infestation, without appendicitis, were younger (9 years (IQR 8, 13) vs. 12 years (IQR 10, 15); *p* = 0.020), had longer duration of symptoms (36 h (IQR, 12, 48) vs. 24 h (IQR, 12, 36); *p* = 0.034), lower body temperature (37 °C (IQR 36.8, 37.4) vs. 37.6 °C (IQR, 37, 38.6) *p* = 0.012), lower incidence of rebound tenderness (*n* = 12; 57.1% vs. *n* = 8; 20%; *p* = 0.003) and less frequent vomiting (*n* = 5; 12.5% vs. *n* = 10; 47.6%; *p* = 0.004) compared to the patients with *Enterobius vermicularis*-associated acute appendicitis. All acute inflammatory markers in the laboratory showed significantly higher values in the group of patients with *Enterobius vermicularis*-related acute appendicitis compared to the patients with *Enterobius vermicularis* infestation, without appendicitis: CRP (*p* = 0.009), WBC (*p* = 0.001) and neutrophils (*p* < 0.001). Interestingly, eosinophilia was not found in any of the groups, although eosinophil counts were statistically significantly higher in children who had *Enterobius vermicularis* infestation than in those with *Enterobius vermicularis*-related appendicitis (2.5%; IQR 0.9, 4.3 vs. 1.8%; IQR 0.7, 2.1; *p* = 0.040). The preoperative characteristics of the children diagnosed with enterobiasis of the appendix, including clinical features and laboratory values, are shown in Table 2.

### 3.3. Histopathological Analysis of Samples with Appendiceal Enterobiasis

Among the samples that were positive for enterobiasis, 21 patients (34.4%) were found to have acute appendicitis on histopathological examination. The majority of cases (*n* = 16; 76.2%) were diagnosed as phlegmonous appendicitis, while the remaining 5 (23.8%) cases were diagnosed as gangrenous appendicitis. No significant inflammatory activity was detected in the other appendiceal samples (*n* = 40; 65.6%). On histopathology, the group of patients with *Enterobius vermicularis*-related acute appendicitis had a larger diameter of the appendix (8.5 mm (IQR 7, 9); vs. 6 mm (IQR 5.5, 7); *p* < 0.001) and less frequently an eosinophilic infiltrate (*n* = 10, 47.6% vs. *n* = 24, 60%; *p* = 0.247) compared to a group with *Enterobius vermicularis* infestation of the appendix. The histopathological analysis of all appendix samples positive for *Enterobius vermicularis* is shown in Table 3.

### 3.4. Outcomes of Treatment

In both groups, almost all cases were assessed in the emergency room because of suspected acute appendicitis. In the majority of cases, an abdominal ultrasound was performed before surgery. In both groups, the majority of patients underwent laparoscopic surgery. The median length of hospital stay was 3 days in both groups. No postoperative complications occurred in the group with *Enterobius vermicularis* infestation, while two complications were recorded in the group with *Enterobius vermicularis*-related acute appendicitis (one surgical site infection treated by incision and drainage and one postoperative ileus due to postoperative adhesions treated by adhesiolysis). No recurrence of the disease was reported in any of the patients. Clinical outcomes of patients with appendiceal enterobiasis are shown in Table 4.

## 4. Discussion

Acute appendicitis is the most common cause of acute abdomen, with an incidence of 1 per 1000 persons per year [32]. Although it can occur at any age, it most commonly affects the pediatric population [2,30]. Although it is one of the most common surgical emergencies, its etiology is still not fully understood [3,33]. Detection of *Enterobius vermicularis* in appendectomy specimens is a relatively rare finding, and several studies have confirmed that *Enterobius vermicularis* may play a role in the pathogenesis of acute appendicitis. The diagnosis is almost never made before surgery, but only after pathohistologic examination of the removed appendix [34].

The aim of this study was to investigate the frequency of *Enterobius vermicularis* findings in appendectomy specimens and to evaluate the clinical and histopathologic features of patients with *Enterobius vermicularis*-associated appendicitis and those with *Enterobius vermicularis* infestation of the vermiform appendix. The incidence of *Enterobius vermicularis* infestation in appendectomy specimens was investigated over 15 years in two large pediatric centers in Croatia, and the study revealed a remarkable but relatively low incidence. This finding suggests that although *Enterobius vermicularis* infection contributes to a subset of cases, it is not prevalent in the pediatric population. The incidence of 0.96% in our study was significantly lower than in the study by Fleming et al., who documented 7.1% of cases with E. vermicularis [12]. This could be due to differences in geographic location or sample size. Hashemi et al. found that among 29,694 appendectomies, only 258 (1%) were positive for *Enterobius vermicularis* [35]. Most of the cases with higher incidence of *Enterobius vermicularis* reported in the literature come from countries with low socioeconomic development. Sah et al. examined 624 surgically removed appendices in Nepal and found *Enterobius vermicularis* in nine (1.62%) cases [36]. In the Gaza Strip, Hamdona et al. detected *Enterobius vermicularis* in 30 (15%) of 200 appendixes on histopathologic examination [37]. In the last decade, several studies conducted worldwide have shown that the infection rate with *Enterobius vermicularis* in children is around 20% [38,39,40]. Rates of up to 38% have been documented in Sri Lanka [41] and as low as 0.21% in Taiwan [42].

The average age of our patients at diagnosis was 11 years, which is consistent with previously published data [12,13]. The higher prevalence of *Enterobius vermicularis* in females (60.7%) is also consistent with the previously known fact that *Enterobius vermicularis* is more common in female patients [43]. As confirmed in previous studies, the majority of patients with *Enterobius vermicularis* infestation had lower levels of acute inflammatory markers, a lower frequency of abdominal symptomatology and low AIR scores [7,44]. In terms of symptoms, several studies confirmed intense itching in the perianal area as one of the most common symptoms, which can lead to insomnia, restlessness and irritability [7,44]. Unfortunately, due to the retrospective design of the present study, these data are not available to us. Zouari et al. found that children with right lower quadrant abdominal pain, anal itching and inflammatory markers in the normal range can be predicted to have *Enterobius vermicularis* infestation [7].

The clinical and histopathologic features of patients with *Enterobius vermicularis*-related acute appendicitis and patients with parasitic infestation of the appendix but without inflammation were quite different. Patients with inflammation had significantly higher levels of inflammatory markers (CRP, WBC, neutrophils). This result differs from a comprehensive literature review by Sousa et al. In their study, there were no significant differences in inflammatory markers between the two groups [16]. Interestingly, in our study, no eosinophilia was detected in any of the groups, which is consistent with previously published studies. Several studies have shown that *Enterobius vermicularis* infection in children is not clinically associated with eosinophilia in peripheral blood, although an association between eosinophilia and helminthic infections is known [7,10,16,45,46]. In contrast, Schroeder et al. reported a correlation of *Enterobius vermicularis* infection and eosinophilia in the peripheral blood of three pediatric patients [47].

The impact of *Enterobius vermicularis* on the development of acute appendicitis remains unclear. Some studies suggest that *Enterobius vermicularis* can cause pain in the right lower abdominal quadrant, but is rarely associated with pathologic findings of acute appendicitis [48]. *Enterobius vermicularis* can be a potential cause of acute appendicitis in children, usually by infection with a high worm burden. This can lead to luminal obstruction, which can cause acute appendicitis. The presence of *Enterobius vermicularis* in the appendix may otherwise only cause local irritation, which may lead to hyperplasia of the lymph nodes but not acute inflammation of the appendix. As confirmed in several publications in the literature, patients with a clinical presentation of acute appendicitis in whom *Enterobius vermicularis* was found in appendix samples had normal inflammatory markers in the laboratory, and only lymphoid hyperplasia was found on histopathologic examination. In these cases, the presence of *Enterobius vermicularis* could in fact only mimic acute appendicitis [21].

In our study, among the samples positive for *Enterobius vermicularis*, 21 patients (34.4%) were found to have acute appendicitis on histopathologic examination. Fleming et al. examined 182 pediatric appendectomy specimens, and similar to our study, there was evidence of acute appendicitis in only 30.8% patients with the presence of *Enterobius vermicularis* [12]. In agreement with our study, Zouari et al. also reported a similar percentage of 43.4% acute appendicitis with the presence of *Enterobius vermicularis* [7].

In conclusion, this study provides valuable insight into the incidence, medical presentation and pathologic aspects of *Enterobius vermicularis*-related appendicitis in pediatric patients. There are several limitations of present study. The retrospective study design could have led to bias and limited the generalizability of the results. In addition, the relatively small number of patients may also influence the results. To fully understand the relationship between *Enterobius vermicularis* infestation and appendicitis, further studies are needed to understand the specific mechanisms.

## 5. Conclusions

*Enterobius vermicularis* was found in 0.96% of surgically removed appendectomy specimens. Pediatric surgeons should always consider *Enterobius vermicularis* infestation as a differential diagnosis when removing appendix vermiformis. Predictive factors such as younger age, longer duration of symptoms, lower body temperature, lower AIR score, lower diameter of the appendix and normal inflammatory markers in the laboratory could help in the diagnosis of *Enterobius vermicularis* infection in the pediatric population presenting with right iliac fossa pain and avoid unnecessary appendectomy.

## Figures and Tables

**Table 1:** Analysis of histopathological specimens (*n* = 6359).

Histopathological Diagnosis	*n* (%)
Acute appendicitis (phlegmonous/suppurative)	3232 (50.83)
Acute appendicitis (gangrenous)	2134 (33.56)
Chronic appendicitis	64 (1.00)
*Enterobius vermicularis*	61 (0.96)
Neuroendocrine tumor	31 (0.49)
Negative appendectomy	837 (13.16)

**Table 2:** Demographic characteristics, preoperative clinical features and laboratory data of patients with appendiceal enterobiasis (*n* = 61).

Variables	Group I (*n* = 21)	Group II (*n* = 40)	*p*
Median (IQR) or *n* (%)	Enterobiasis + AA	Enterobiasis	
Demographic characteristics of patients			
Age (years)	12 (10, 15)	9 (8, 13)	0.020 *
Gender			0.074 ^†^
Male	12 (57.1)	12 (30)	
Female	9 (42.9)	28 (70)	
Weight (kg)	42 (38, 62)	40 (30, 50)	0.114 *
Height (cm)	157.5 (148, 169.5)	147 (136, 162.5)	0.018 *
BMI (kg/m^2^)	18.2 (16.4, 21.5)	18.1 (16.1, 19.8)	0.756 *
Clinical features of patients			
Duration of symptoms (h)	24 (12, 36)	36 (12, 48)	0.034 *
Body temperature (°C)	37.6 (37, 38.6)	37 (36.8, 37.4)	0.012 *
Pain in RLQ	20 (95.2)	37 (92.5)	>0.999 ^‡^
Nausea	11 (52.4)	14 (35)	0.273 ^†^
Vomiting	10 (47.6)	5 (12.5)	0.004 ^‡^
Rebound tenderness	12 (57.1)	8 (20)	0.003 ^†^
AIR score	7 (5, 9.5)	3 (2, 5)	<0.001 *
Laboratory preoperative parameters			
C-reactive protein (mg/L)	32.1 (8.1, 63.2)	5.2 (1.1, 22.4)	0.009 *
White blood cells (×10^9^/L)	16.9 (12.8, 19.3)	10.9 (8.7, 14.1)	0.001 *
Neutrophils (%)	83.2 (77.5, 87.8)	73.5 (60, 79.3)	<0.001 *
Eosinophils (%)	1.8 (0.7, 2.1)	2.5 (0.9, 4.3)	0.040 *
Basophils (%)	0.3 (0.2, 0.5)	0.4 (0.3, 0.5)	0.912 *
Lymphocytes (%)	11.2 (6.7, 13.4)	13.9 (12, 25.8)	0.035 *
Monocytes (%)	5.9 (4.1, 7.3)	7.7 (5.3, 10.1)	0.004 *

* Mann–Whitney U-test; ^†^ Chi-squared test; ^‡^ Fisher’s exact test; data presented as median (IQR) or *n* (%); AA—Acute appendicitis; IQR—Interquartile range; BMI—Body mass index; RLQ—Right lower quadrant; AIR—Appendicitis inflammatory response.

**Table 3:** Histopathological features of samples with appendiceal enterobiasis (*n* = 61).

Variables	Group I (*n* = 21)	Group II (*n* = 40)	*p*
Median (IQR) or *n* (%)	Enterobiasis + AA	Enterobiasis	
Diameter of appendix (mm)	8.5 (7, 9)	6 (5.5, 7)	<0.001 *
Length of appendix (cm)	7.5 (6.5, 8.5)	7 (6.5, 8)	0.541 *
Inflammatory infiltrate present	21 (100)	1 (2.5)	<0.001 ^†^
Eosinophilic infiltrate present	10 (47.6)	24 (60)	0.247 ^‡^
Fecalith present	5 (23.8)	7 (17.5)	0.735 ^†^

* Mann–Whitney U-test; ^†^ Fisher’s exact test; ^‡^ Chi-squared test; AA—Acute appendicitis; IQR—Interquartile range.

**Table 4:** Clinical outcomes of patients with appendiceal enterobiasis (*n* = 61).

Variables	Group I (*n* = 21)	Group II (*n* = 40)	*p*
Median (IQR) or *n* (%)	Enterobiasis + AA	Enterobiasis	
Reason for appendectomy			>0.999 *
Acute appendicitis	21 (100)	39 (97.5)	
Incidental appendectomy	0 (0)	1 (2.5)	
Type of surgery			0.575 ^†^
Laparoscopic appendectomy	12 (57.1)	27 (67.5)	
Open appendectomy	9 (49.2)	13 (32.5)	
Abdominal ultrasound performed	14 (66.7)	33 (82.5)	0.162 ^†^
Length of hospital stay (days)	3 (3, 5.5)	3 (2, 4.5)	0.406 ^‡^
Complications	2 (10)	0 (0)	0.107 *

* Fisher’s exact test; ^†^ Chi-squared test; ^‡^ Mann–Whitney U-test; AA—Acute appendicitis; IQR—Interquartile range.

## Data Availability

The data assessed and reported here can be obtained from the authors upon reasonable request and following ethical and privacy principles.
